# 3D printed inserts for reproducible high throughput screening of cell migration

**DOI:** 10.3389/fcell.2023.1256250

**Published:** 2023-08-30

**Authors:** Abhayraj S. Joshi, Mukil Madhusudanan, Ivan Mijakovic

**Affiliations:** ^1^ The Novo Nordisk Foundation Center for Biosustainability, Technical University of Denmark, Kongens Lyngby, Denmark; ^2^ Department of Biology and Biological Engineering, Division of Systems and Synthetic Biology, Chalmers University of Technology, Gothenburg, Sweden

**Keywords:** *in vitro* studies, cell migration, cell migration assay, scratch assay, wound healing assay, 3D printing, biocompatible cell inserts, image analysis

## Abstract

Cell migration is a fundamental and complex phenomenon that occurs in normal physiology and in diseases like cancer. Hence, understanding cell migration is very important in the fields of developmental biology and biomedical sciences. Cell migration occurs in 3 dimensions (3D) and involves an interplay of migrating cell(s), neighboring cells, extracellular matrix, and signaling molecules. To understand this phenomenon, most of the currently available techniques still rely on 2-dimensional (2D) cell migration assay, also known as the scratch assay or the wound healing assay. These methods suffer from limited reproducibility in creating a cell-free region (a scratch or a wound). Mechanical/heat related stress to cells is another issue which hampers the applicability of these methods. To tackle these problems, we developed an alternative method based on 3D printed biocompatible cell inserts, for quantifying cell migration in 24-well plates. The inserts were successfully validated via a high throughput assay for following migration of lung cancer cell line (A549 cell line) in the presence of standard cell migration promoters and inhibitors. We also developed an accompanying image analysis pipeline which demonstrated that our method outperforms the state-of-the-art methodologies for assessing the cell migration in terms of reproducibility and simplicity.

## 1 Introduction

Cell migration is a fundamental phenomenon associated with a vast range of basic physiological processes (such as immune cell migration), developmental processes (such as neuronal cell migration) as well as in non-communicable diseases (such as cancer) (Yamada and Sixt, 2019; SenGupta et al., 2021; Lusby et al., 2022; Merino-Casallo et al., 2022; Pawluchin and Galic, 2022). The cells respond to various chemical and biophysical cues that originate internally or externally and migrate in single cell or collective fashion (SenGupta et al., 2021; Lusby et al., 2022; Merino-Casallo et al., 2022; Pawluchin and Galic, 2022). Over the past decade many successful attempts have been made to unravel cell migration phenomenon with respect to cell behavior during migration and molecular players involved in it using experimental and simulation techniques (Grada et al., 2017; Haridas et al., 2018; Matsiaka et al., 2019; Melo et al., 2021). Especially in cancer, understanding cell migration is very important because of its principal role in metastasis. In cancer metastasis, the cell migrates away from primary tumor site and enters blood circulation (intravasation) and further migrates to secondary tissues (extravasation) to create secondary tumors, which increase complications by several orders of magnitude. Thus, metastasis is a leading cause of deaths in cancer patients (Stuelten et al., 2018; Lusby et al., 2022; Merino-Casallo et al., 2022). Therefore, study of cell migration has received prime importance in the perspective of diagnosis as well as treatment of cancer.

Apart from few exceptions such as epithelial cell migration involved in skin wound closure and cell migration on bones, all cell migration phenomena occur in 3 dimensions (3D) in normal physiological setup (Yamada and Sixt, 2019). Surrounding extracellular matrix (ECM) and neighboring cells have direct impact on the mode and rate of cell migration (Stuelten et al., 2018; Matsiaka et al., 2019; Yamada and Sixt, 2019; SenGupta et al., 2021; Lusby et al., 2022; Merino-Casallo et al., 2022; Pawluchin and Galic, 2022). In recent years, the cell migration studies have seen few technical or experimental advances such as study of cell migration in 3D environment (e.g.,: in Matrigel™ support) (Audoin et al., 2022) or in soft or hard biocompatible hydrogels (Trappmann et al., 2017), cell migration in spheroids (Zhang et al., 2022), and cell migration in microfluidic devices (Schwarz et al., 2016; Shih et al., 2019). As it is difficult to mimic 3D cell migration in laboratory setup, this phenomenon is most often studied in an *in vitro* 2-dimensional (2D) cell cultures (Riahi et al., 2012; Stamm et al., 2016). 2D studies provide conceptual evidence and understanding of the cell migration rate and directionality (Riahi et al., 2012; Stamm et al., 2016; Grada et al., 2017; Boyer et al., 2018; Acosta et al., 2022), but significant differences exist between 2D and 3D cell migration, so caution must be exerted when interpreting 2D migration results. 2D cell migration studies are often associated with absence and presence of additional chemical entities such as cell migration promoters (e.g.,: growth factors) or cell migration inhibitors (e.g.,: potential anticancer drug candidates). 2D cell migration assay (also known as scratch assay or wound healing assay) can be performed by various techniques as mentioned in Table 1 (Riahi et al., 2012; Stamm et al., 2016). Each technique has its own advantages and disadvantages. The common factor involved in these techniques is cell scraping (mechanical, laser- or electric current-assisted) which harms the cellular structure and associated ECM. Neighboring surviving cells may also experience mechanical or heat related stress. Such stress could induce a response from cells leading to changes in basic cellular physiology, which obscure the results obtained in these assays (Riahi et al., 2012; Stamm et al., 2016). In order to prevent the changes in cellular physiology, ibidi GmbH (Germany) has developed specialized inserts, essentially blocks of polymer, that create a cell-free area (similar to a scratch or a wound) without impacting basic cellular physiology (Reference: https://ibidi.com/content/category/21-wound-healing-and-migration (Last accessed on 26/12/2022)). These inserts work on the principle of putting up a physical barrier that prevents formation of a cell monolayer in a specific area. However, the cost of such inserts is very high. Also, these inserts are meant for single use in an experiment. Thus, in an attempt to improve this experimental technique, other types of inserts have been introduced, which work on the principle similar to the ibidi GmbH inserts. The most recent advance in such inserts were reported by Acosta *et al.* and Boyer *et al.* Even though their inserts yielded reproducible results, the technology still has some notable limitations, mentioned in Table 2 (Boyer et al., 2018; Acosta et al., 2022).

**TABLE 1:** Various 2D methods that are reported in the literature for carrying out cell migration studies, listed with their main advantages and disadvantages.

Method	Description	Advantage	Disadvantage
Scratch Assay	Manually scratching confluent monolayer with pipette tip or similar pointy object	1. Easy to use	1. Irregular scratches
		2. Suitable for widely available culture conditions and equipment	2. Destruction of underlying matrix substrate coating and extracellular matrix secreted by cells
			3. Accumulation and poor removal of loosened cells on the edges of the scratch
Stamping	Application of pressure on cells in a defined area	1. Wound of any shape possible	1. Irregularities in manual pressure applied to stamp which could lead to reduced reproducibility
		2. Influence on cell debris on migration can be monitored	
		3. Cell culture matrix coating remains intact	
		4. Combination with thermal wounding possible	
Thermal wounding	Application of excessive heat to a restricted area of a confluent cell layer	1. Possibility of studying thermo-mechanical damage	1. Heat might not be restricted to a certain area
			2. Heat transfer to nearby cells
			3. Heat development can affect cell viability
Electrical wounding via Electrical cell substrate impedance sensing (ECIS)	Application of high voltage pulse to a restricted area	1. Measurement and control of cell destruction and regrowth by impedance	1. Difficult to detach/destruct the confluent cell layer of keratinocytes and fibroblast; thus, not suitable to all cell types
		2. Real time measurement	2. Changes in adhesion and cell density after impedance alter measurement
		3. Automatic elimination of human errors	3. Low throughput
		4. Quantitative and highly reproducible method	4. Specialized equipment is need which increases cost of experimentation
			5. Skilled personnel are needed to carry out experiment
Optical wounding/Laser wounding	Creates a wounded area with a laser	1. High reproducibility	1. Heat development can affect cell viability
		2. High throughput	2. Heat might not be restricted to a certain area
		3. Sterile environment	3. Acquisition of Laser Enabled Analysis and Processing (LEAP) instrument necessary

**TABLE 2:** The most recent 3D printed inserts developed for studying cell migration, listed with their main advantages and disadvantages.

Device for cell migration study	Description	Advantages	Disadvantages
Lab-made 3D printed stoppers	Designed using CAD modeling through Autodesk Inventor (AutoDesk, United States of America) Individually printed stoppers with end part having circular shape to create circular cell-free area	1. Low 3D printing cost	1. Cell-free area of only circular shape
		2. Simple design for putting in a single well	2. No autoclavable material
		3. No need of external stabilizer clamps	3. Stoppers only for 96-well plates
			4. No possibility for cell monolayer visualization when inserts are placed in wells along with cells
3D printed cell exclusion spacers (CES)	Designed using TinkerCAD (AutoDesk, United States, A squared mesh like design with end part of various geometrical shapes (rectangle, circle, etc.) to create cell-free area)	1. Cell-free area with shape of any geometrical shape such as rectangle, circle, plus sign	1. No autoclavable material
		2. CES fit for multiple well plates (6-well, 12-well, 24-well, 96-well plates)	2. Need of special 3D printed external stabilizer clamps to hold CES in place
		3. Possibility for cell monolayer visualization when CES are places in wells along with cells	3. High 3D printing cost Bulky design

**FIGURE 1:**
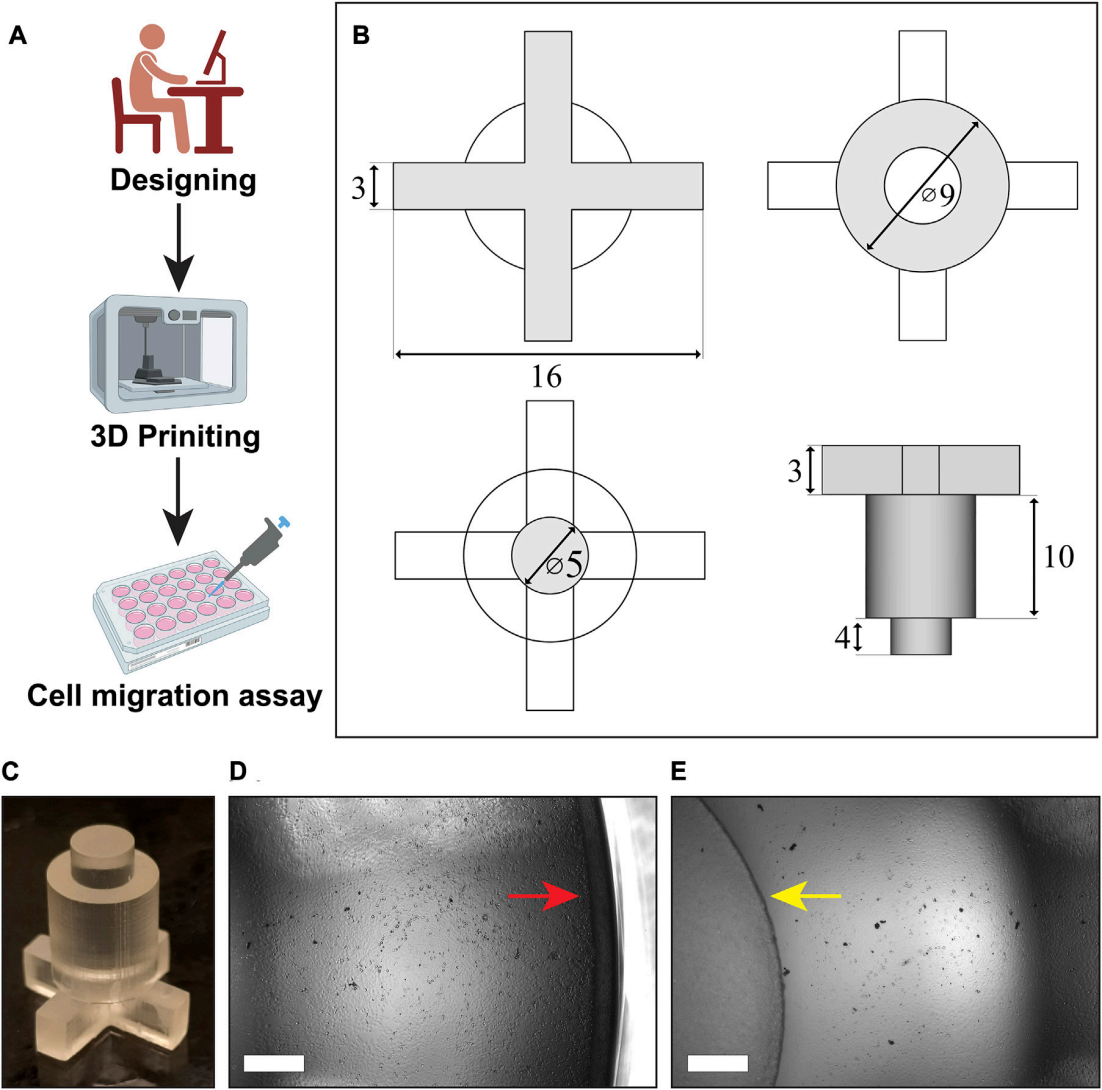
Designing, 3D printing and use of cell inserts in cell migration assay: **(A)** Schematics showing overview of the whole process, **(B)** the blueprint of our inserts designed in FreeCAD software, **(C)** the cell insert obtained after 3D printing, **(D,E)** visualization of the cell monolayer between the well border (red arrow) and the insert (yellow arrow), (Scale bar in the images = 500 µm).

In order to overcome these limitations of inserts for 2D cell migration assays, we designed and 3D printed an alternative novel cell insert with a characteristic shape (Figure 1) that would allow reproducible creation of cell-free area, easy and hassle-free usage with possibility of imaging while insert is placed in well. To validate our inserts, we carried out an *in vitro* cell migration assay in cell culture medium supplemented with cell migration promoter (epidermal growth factor (EGF)) (Kim et al., 2015), a cell migration inhibitor (colchicine) (Wang et al., 2019), and an anticancer drug (doxorubicin hydrochloride) (Thorn et al., 2011). For bulk image analysis we also developed a new image analysis pipeline. Our overall results depict a modified, novel, and improved design for cell inserts and its applicability in high throughput study of cell migration in 2D cell culture setup with high reproducibility.

## 2 Materials and methods

### 2.1 Materials

The high temperature resin (Catalog no. RS-F2-HTAM-02) as well as the printer (Form3) used for 3D printing of inserts were purchased from Formlabs (Europe). 2-propanol solvent (Technical grade, Catalog no. 20922.320) used for washing the 3D printed inserts were purchased form VWR (Part of Avantor), Denmark. All the cell culture supplies such as Dulbecco’s minimum essential medium (DMEM) with F12 Ham’s mixture (1:1 ratio) (Catalog no. D8062-500ML), penicillin-streptomycin mixture (Catalog no. P4333-100ML), Dulbecco’s phosphate buffered saline (DPBS) (Catalog no. D8537-6X500ML), colchicine (>95% Purity, HPLC grade) (Catalog no. C9754-100MG), and doxorubicin hydrochloride salt (Pharmaceutical standard, reference material grade) (Catalog no. PHR1789-200 MG) were purchased form Sigma Aldrich (Merck), Denmark. Fetal bovine serum (Catalog no. 10500064) and Epidermal growth factor (EGF) (Catalog no. PHG0313) was purchased from Life Technologies, ThermoFisher Scientific, Denmark. Mycozap antibiotic (Catalog no. VZA-2011) was purchased from Lonza (Bionordika, Denmark). All cell culture vessels such as flasks, well-plates were purchased from Corning Inc., Denmark. Human lung adenocarcinoma cell line (A549 cell line) (Catalog no. CCL-185) was purchased from American Type Culture Collection (ATCC, Germany headquarters).

### 2.2 Methods

#### 2.2.1 Designing and 3D printing the cell culture inserts

FreeCAD software (Version 0.20.1) was used to design the cell inserts. The insert was designed to have three distinct parts: top layer having “plus design”, middle layer having “bigger cylindrical design”, and bottom layer having “smaller cylindrical design” (Figures 1A–C). The top section with plus design was 16 mm in length and 3 mm in height. Such design was made to allow for easy insertion of pipetting tips. The bottom cylindrical section having height of 4 mm was designed for creating the cell-free area with a diameter of 5 mm. The top section and the bottom section were supported by a bigger cylinder in the middle with a diameter of 9 mm and 10 mm height. The overall height of inserts was kept to 17 mm, compatible with closure of the 24-wells plate lid. The *.STL file for 3D printing is provided as Supplementary File (Supplementary Material).

#### 2.2.2 Cell culture

For all cell culture experiments, human lung cancer cell line: A549 (CCL-185™) was used. Unless and otherwise mentioned, the DMEM with F12 Ham’s mixture (1:1) supplemented with 10% fetal bovine serum, 1x penicillin-streptomycin, and 1x Mycozap antibiotics was used as growth medium in all the experiments. All experiments were carried out in a humidified incubator maintained at 37°C with supply of 5% carbon dioxide gas.

#### 2.2.3 Cell viability

In order to assess the effect of resin used to make the inserts on cell viability, cells were trypsinized and seeded at seeding density of 2×10^4^ cells/well in a 24-well plate containing the inserts. After incubation for 24 h, the inserts were removed. Then, old medium was removed and DMEM supplemented with Cell Counting Kit-8 (CCK-8) reagent was added to all wells. CCK-8 dye concentration was kept as per manufacturer’s protocol (10 µL reagent per 100 µL of DMEM). After incubating cells for 1 h (37°C and 5% carbon dioxide gas), the color intensity was measured at 450 nm using plate reader. The cells grown without the inserts served as a control for this experiment.

#### 2.2.4 Cell migration assay

The cell migration assay was performed in 24-well plates. First, the inserts were sterilized by autoclaving (CertoClav, Buch and Holm, Denmark). After sterilization, the inserts were placed in a sterile 24-well plate. The A549 cells grown in T75 flask were trypsinized and seeded in the 24-well plate containing the inserts with seeding density of 2 × 10^5^ cells/well. Then, cells were incubated for a time period of 24 h, within which they formed confluent monolayer inside the well around the insert (Figures 1D, E). Next, the inserts were removed and the A549 cell monolayer was gently washed twice with DPBS to remove dead cells and traces of the old medium. After washing, the cells were separately treated with DMEM, DMEM +10% FBS, DMEM +10%FBS +10 ng/mL EGF, DMEM +10%FBS +2.5 μg/mL Colchicine, and DMEM +10%FBS +1 μg/mL Doxorubicin (henceforth termed as DMEM, FBS, EGF, Colchicine, Doxorubicin treatments for simplification) to analyze cell migration under the influence of promoters and inhibitors. Cells treated with DMEM with and without serum served as a control against remaining groups in this experiment. After addition of DMEM with supplements, the 24 well plate was immediately placed in an incubator mounted with IncuCyte S3 (Firmware 20192.4.0.0, GUI Version 2019B Rev2, and Controller Version 2019B Rev 3). The program was set for image capture of the whole well (maximum zoom level 4X) for 3 days with a 3 h interval. The images were collected in 24-bit TIF format.

#### 2.2.5 Image analysis

The images collected from IncuCyte S3 were processed using FIJI (Fiji Is Just ImageJ, Version 1.53t, National Institutes of Health, USA, Java 1.8.0_322 (64 bit), https://imagej.nih.gov/) in ‘batch processing’ format. To do so, we developed a macro script for converting RGB images to binary images for highlighting and measuring the cell-free area in each image. This macro script is provided in Supporting Material. Briefly, each image was first converted to 8-bit grayscale format and then cropped to focus on cell free area created by the inserts. Then by using “Find Edges” option, cell borders were highlighted. After adjusting the threshold (with “MaxEntropy” option under “Dark background”) and using ‘Invert LUT’ option, the images were converted to binary images. To highlight the cell-free area in binary images, “Erode” and “Fill holes” options were used. Then, using original scale of images (0.3525 pixels/µm), the absolute areas were calculated. From this, percentage areas were calculated and plotted against time to get the cell migration kinetics. Based on the same data the effect of treatment by various cell migration promoting and inhibiting agents (EGF, colchicine, and doxorubicin) was analyzed.

#### 2.2.6 Statistical analysis

Statistical analysis was done using GraphPad Prism software (Version 9.4.0 (673), GraphPad LLC). All the experiments were done in triplicate and results were reported as mean ± standard deviation unless and otherwise stated. For analyzing percentage of cell viability within two groups (the inserts treated cells and untreated control cells), paired *t*-test was used. Further, to analyze the cell-free area created by various inserts in 24-well plates, one-way ANOVA test was used. Post-hoc tests were also used along with one-way ANOVA to confirm the statistical findings in multiple comparisons. For analyzing the effect of various treatments on cell migration at each time point, two-way ANOVA test with multiple comparisons (with “Treatment” as column variable and “Time” as row variable) was used. All the statistical tests were performed at confidence interval of 95% (i.e., with level of significance of 0.05). The data, for which statistical analysis showed *p*-value lesser than 0.05, were considered significantly different from each other.

## 3 Results

### 3.1 Novel design of our 3D printed cell inserts offered easy hassle-free usage

The design of the insert was made using an open source “FreeCAD” software in order to obtain the cell insert suitable for a 24-well plate (Figures 1A–D). With the dimensions given in methodology section, cell inserts were printed using Formlab Form 3 printer with high temperature resin that has heat deflection temperature (HDT) of 238°C at 0.45 MPa. These inserts were made cell culture ready within time period of about 9 h which included 1) designing for 1 h, 2) printing for 5 h, 3) washing for 6 min, 4) curing for 2 h, and 5) autoclaving for 15 min. As shown in Figure 1, these inserts have a top portion with shape of “plus sign”, a middle portion with shape of “larger cylinder”, and finally a bottom portion with shape of “smaller cylinder”. The ‘plus sign’ design at the top created a four-quadrant space that enabled us cell visualization in real time via brightfield microscopy, while the insert is kept in the well plate. It also allowed us to replace the cell culture medium over the course of an experiment. The middle “larger cylinder” design connected top portion and bottom portion and added weight to the whole insert that was necessary for keeping it inside well for creating cell-free area. The bottom ‘smaller cylinder’ touching to the bottom of well-plate created the circular cell-free area where collective cell migration was analyzed. Overall design had height of 17 mm which is exactly same with that of well. Owing to the length of “plus design” part (16 mm), the inserts were fit perfectly inside the well. This prevented their horizontal movement in the well helping the creation of uniform cell-free area.

### 3.2 The material of our inserts did not affect cell viability

**FIGURE 2:**
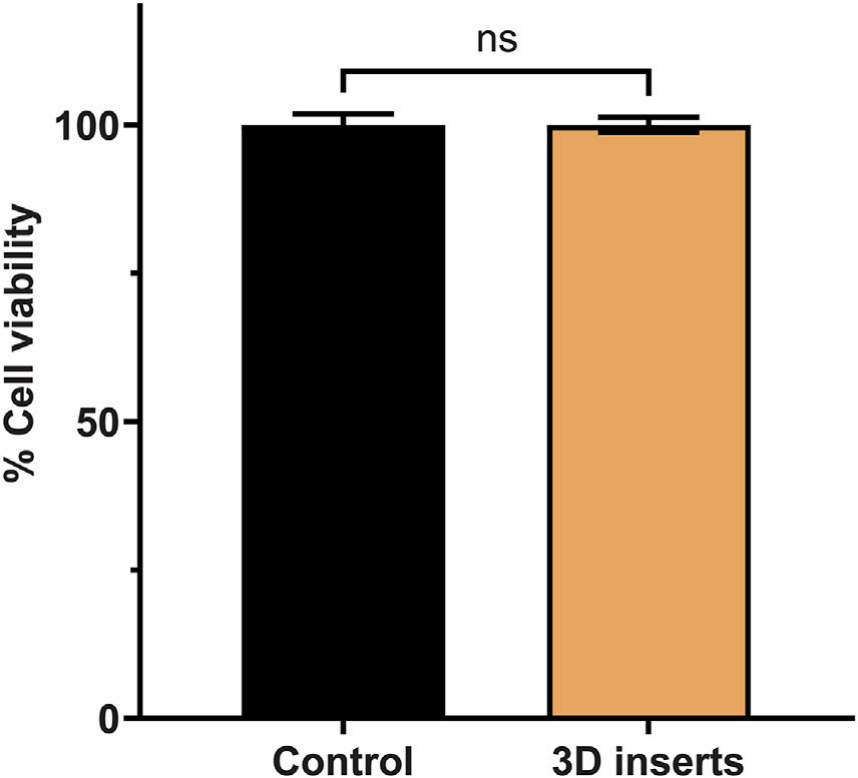
Cell viability of A549 cells in the absence (control) and presence of the cell inserts.

After printing, washing with isopropanol, and curing the cell inserts under UV light (for 2 h), they were subjected to 3 cycles of washing with sterilized water and then to autoclaving to make them useable in cell culture experiments. First, we addressed the question of any potential effects of the insert material on cell viability. A stable cell monolayer around these inserts is the primary requirement for cell migration study and hence, after seeding, cells are expected to grow, divide, and proliferate to form a monolayer in the presence of these inserts. In order to validate the inserts, we selected pulmonary adenocarcinoma cells (A549 cell line) that were isolated form a 58 years old male patient (Reference: https://www.atcc.org/products/ccl-185 (Last accessed on 26/12/2022)). This cell line represents the most commonly occurring non-small cell lung cancer (NSCLC). We first checked A549 cell viability with CCK-8 cell viability kit as per manufacturer’s instructions. We observed 100% ± 4.5% viability for control group and 100% ± 3.1% viability for A549 cells grown in presence of the inserts. As shown in Figure 2, no significant differences were found in the percent cell viability for both control and the insert treated groups (paired *t*-test, *p*-value >0.05). This proved that our inserts are perfectly compatible with A549 cells.

### 3.3 Cell free area created by the inserts is uniform and reproducible

**FIGURE 3:**
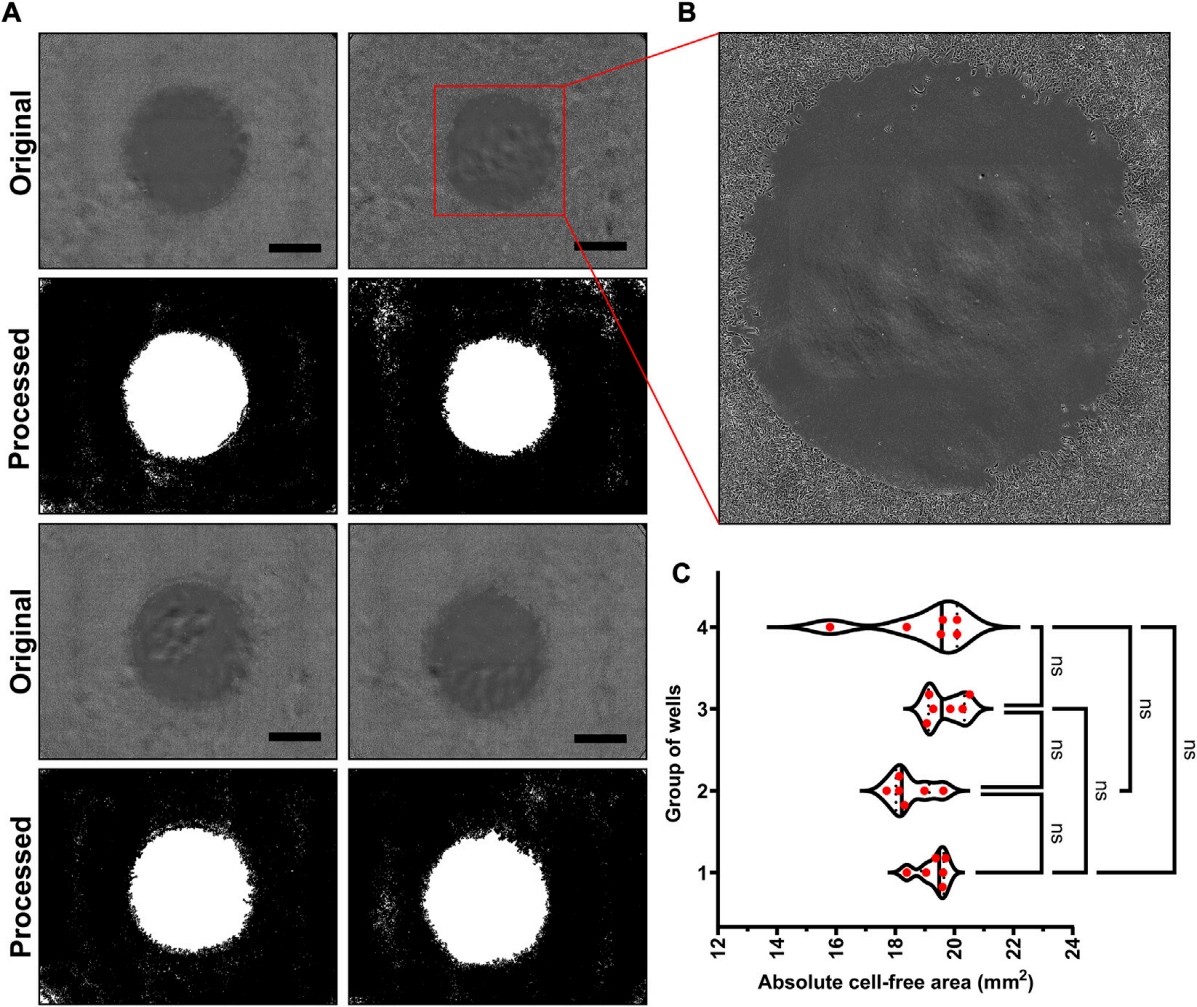
Reproducibility of our novel cell inserts: **(A)** The representative bright field images showing cell-free areas created by the inserts and their binary counterparts obtained after image analysis showing white cell-free area and black area filled with cell monolayer, **(B)** The zoomed-in cell-free area of one well that shows no leakage under cell insert, and **(C)** The absolute areas calculated from the images showing same cell-free areas for 24 inserts. (Scale bar in images = 1000 µm).

The success of any assay lies in its capacity to produce reproducible results, and in case of cell migration assay, this reproducibility is denoted by the uniform cell-free area in each well. As shown in Figure 3, we found that, the inserts created uniform cell-free area in reproducible fashion. The representative bright field images and binary images obtained after image analysis for cell-free areas are provided in Figure 3A. As shown in Figure 3B, the absolute areas for all cell inserts remained in the range of 19.1 ± 1.02 mm^2^, which is very close to the theoretical area (19.6 mm^2^). No statistically significant difference was observed when 6 replicates (randomly selected and divided in 4 groups) were analyzed and tested using one-way ANOVA test (*p*-value >0.05). Furthermore, as the bottom cylindrical part is touching the surface of the well-plate, we found no cells in the cell-free area at first time point just before the start of treatment. This proved that the inserts are not leaky. These all findings confirm that all inserts were printed uniformly and created the same cell-free area in all the wells. With these findings (Figures 2, 3), we next sought to check cell migration under the influence of various treatments.

### 3.4 Applicability of the inserts was validated by performing collective cell migration assay in presence of different treatments

To assess whether our novel inserts can overcome the limitations of currently available devices and methods (Tables 1, 2), we performed a cell migration study with various compounds that promote and inhibit cell migration. After creating a monolayer in a 24-well plate in presence of the inserts, they were removed. After gently washing the monolayer with DPBS, the monolayer was treated with DMEM (only medium), DMEM +10% FBS (medium with serum supplement), DMEM +10% FBS + EGF (medium with serum and growth factor supplements), DMEM +10% FBS + colchicine (medium supplemented with serum and microtubule binding alkaloid), and DMEM +10% FBS + doxorubicin (medium supplemented with serum and a known FDA approved anticancer drug) (henceforth termed as DMEM, FBS, EGF, Colchicine, Doxorubicin treatments for simplification). Among these, FBS and EGF treatments are expected to enhance cell migration; whereas colchicine and doxorubicin treatments are expected to inhibit it. The details of their mechanism have been mentioned in discussion section.

**FIGURE 4:**
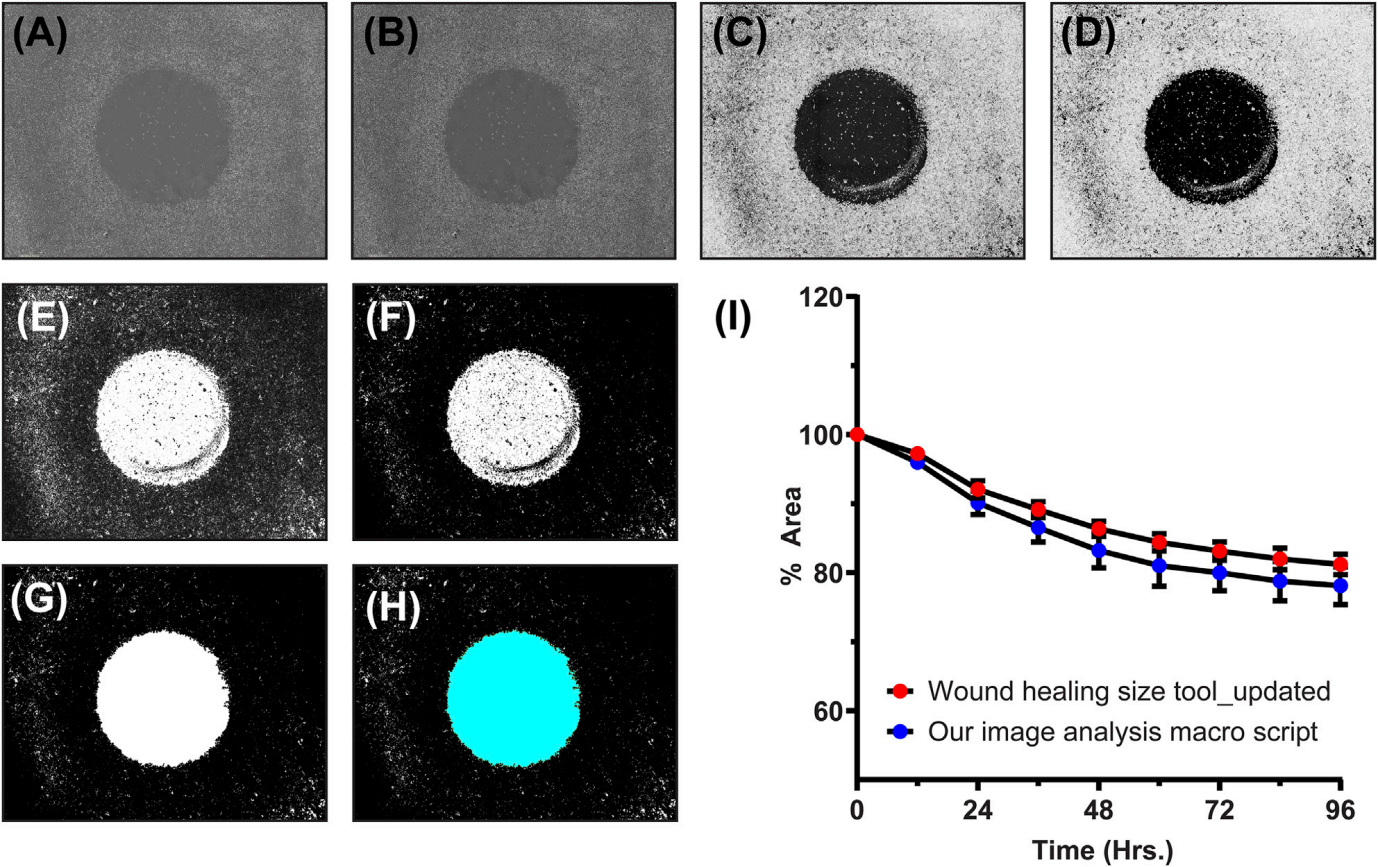
An example of our newly developed image analysis macro script: **(A)** Original RGB image, **(B)** converted 8-bit grayscale image, **(C)** image with highlighted cellular structures, **(D,E)** binary image created at particular threshold, **(F)** eroded binary image, **(G)** binary image with filled holes, **(H)** final image for cell-free area calculation (cyan colored), and **(I)** the line graph showing comparison of migration kinetics calculated using our image analysis macro script and one of the standard ImageJ analysis program (Wound healing size tool_updated).

After treatment, the imaging was done using automated imaging program with the help of IncuCyte S3 live cell analysis instrument. For obtained images, the analysis was done using FIJI (Fiji Is Just ImageJ, Version 1.53t, National Institutes of Health, USA, Java 1.8.0_322 (64 bit)). For cell migration analysis, several Matlab (DuChez, 2018; Zabary and Zaritsky, 2022), Python (Tsai et al., 2019; Garcia-Fossa et al., 2020), and Java (Cordelières et al., 2013; Masuzzo et al., 2017) based programs are available. The use of these programs requires expertise in programing languages and some of them are time consuming for thousands of images obtained from high throughput experiments. Thus, to simplify the image analysis, we developed a macro script (Supplementary Material: Supplementary Method S1) which first converts all the RGB images (Figure 4A) to 8-bit grayscale images (Figure 4B). Then, depending on the magnification of the images and objective of microscope (4X—standard objective set for whole well scan), a global scale was set (0.3525 pixels/µm). Next, using the “Find Edges” option, the cellular structures were highlighted (Figure 4C). By setting auto threshold (MaxEntropy option) with black background and using the “Invert LUT” option, binary images were obtained (Figures 4D, E). Treating the binary images with “Erode” and then with “Fill Holes” option, the cell-free area was highlighted (Figures 4F, G). Then, using “Analyze Particles”, the cell-free area was calculated (Figure 4H). A fixed range of 100000-700000000 was used for measuring cell-free areas in all the images. From these absolute areas, percentage areas were calculated, and these were plotted against time to represent the kinetics of cell migration. In order to check the accuracy of our macro script, we also analyzed one set of images using already available ImageJ plugin called “Wound healing size tool (updated)”. Figure 4I represents the comparison of migration kinetics for cells grown in DMEM supplemented with FBS. No significant difference (*p*-value >0.05, *t*-test) was observed in kinetics obtained from both image analysis program. This suggests that our simplified macro script analyze the images in same reproducible and accurate manner yielding equally comparable results. By putting this newly developed script in “Batch Process” option of FIJI, one can analyze thousands of images correctly under universal parameter definitions within a short period of time.

**FIGURE 5:**
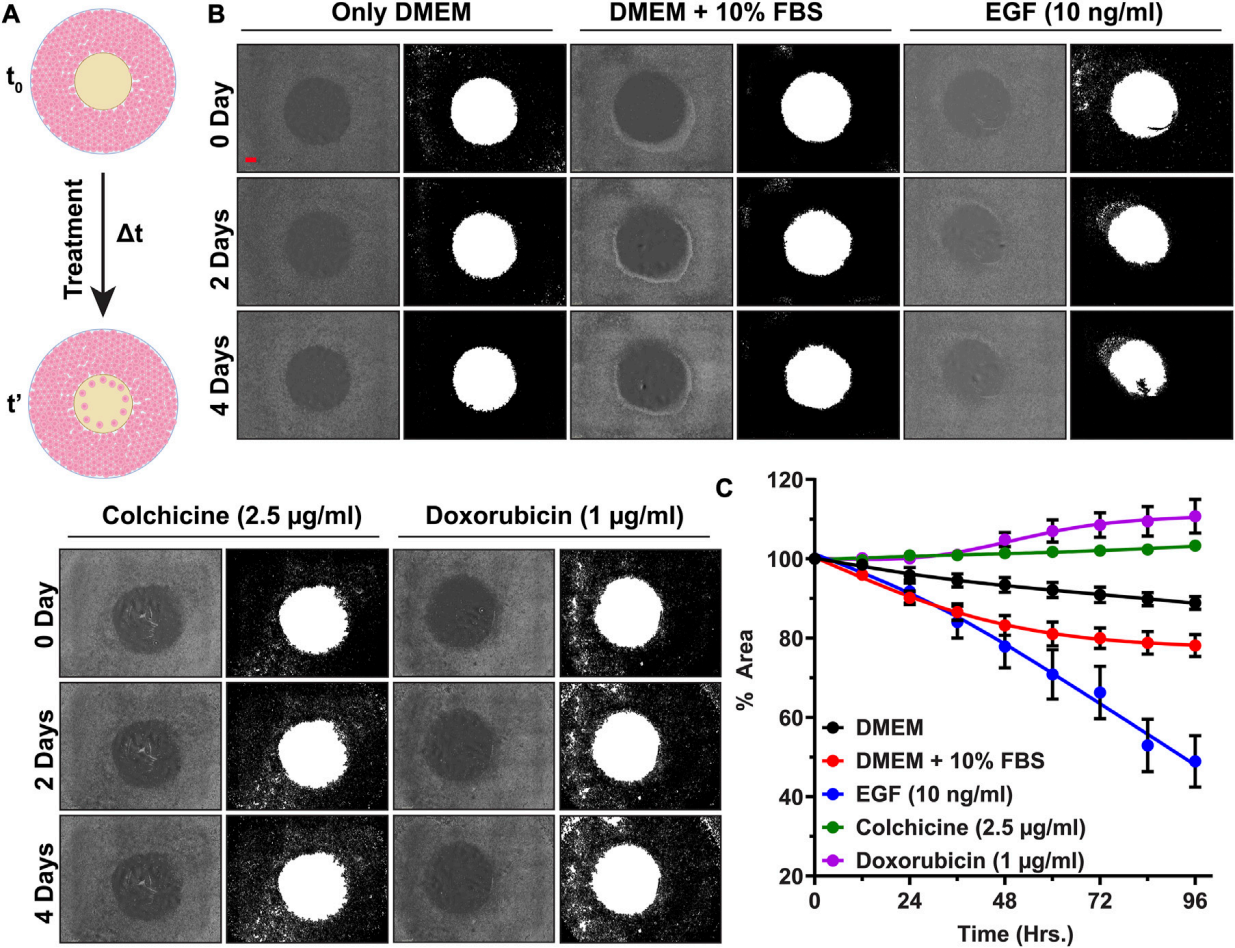
High throughput cell migration assay for validation of our cell inserts: **(A)** Schematics showing cell migration assay under influence of various treatments over time period Δt, **(B)** Representative original bright field images of cell-free areas and their binary counterparts showing cell migration progress at 0th, 2nd, and 4th day for A549 cells treated with DMEM, FBS, EGF, Colchicine, and Doxorubicin, **(C)** The line graph representing % cell-free area ±SEM of three replicates, (Scale bar in first image (red-colored bar) = 800 µm).

After checking the accuracy of the newly developed image analysis script, we analyzed images belonging to aforementioned treatments. The differences in the kinetics of cell migration under the influence of various treatments (DMEM, FBS, EGF, colchicine, and doxorubicin) are shown by line graph in Figure 5. From graph, it can be deduced that supplementing the medium with 10% FBS had a positive impact on cell migration. After 96 h, the cell-free area for DMEM alone was 88.8% ± 2.8%, whereas for FBS treatment, it was 78.1% ± 4.7%. For EGF treatment, the cell-free area after 96 h was only 48.9% ± 11.2%. Two inhibitors of cell proliferation and migration, colchicine and doxorubicin, were also used in our assay. As shown in Figure 5, with these inhibitors we did not observe any significant reduction in the cell-free area within the duration of our experiment. After 96 h, the cell-free areas for colchicine and doxorubicin treatments were found to be 103.3% ± 0.4% and 110.7% ± 7.4%, respectively. These findings are in corroboration with previously published data (Thorn et al., 2011; Bhattacharya et al., 2016; Zhang et al., 2019; Oh et al., 2022). Then, we also sought to determine cell movement and average cell velocity using CellTracker program (Piccinini et al., 2015) (Supplementary Material: Supplementary Method S2, Supplementary Figure S1). The average cell velocities for respective treatments have been shown in Table 3. As expected, the cell velocity was the highest for EGF treated cells and the lowest for colchicine and doxorubicin treated cells. This CellTracker data showed same trend and corroborated well with cell migration kinetics results (Table 3; Supplementary Figure S1).

**TABLE 3:** The average cell velocities calculated for 10 representative randomly selected cells using CellTracker software for each treatment.

Sr. No.	Treatment given to cells	Average cell velocity (µm/h)
1	DMEM	0.15 ± 0.03
2	FBS	0.52 ± 0.13
3	EGF	0.69 ± 0.19
4	Colchicine	0.04 ± 0.01
5	Doxorubicin	0.05 ± 0.03

Furthermore, two-way ANOVA analysis as explained in Supplementary Material indicated that during the initial 24 h no significant cell migration changes (in terms of filling up the cell-free area by cell proliferation and cell migration) were observed for any treatment groups. After 48 h, a rapid decrease in % cell-free area denotes synergistic action of EGF along with 10% FBS; whereas no decrease in % cell-free area depicts complete inhibition of cell migration by colchicine and doxorubicin (Supplementary Material: Supplementary Method S3, Supplementary Figure S2). This suggests that the cells were proliferating and migrating with different rates under the influence of given treatment. Finally, the morphological inspection of A549 cells under given treatments (Supplementary Material: Supplementary Method S4, Supplementary Figure S3) revealed healthy normal epithelial morphology in DMEM, serum supplemented DMEM, and EGF supplemented DMEM. In contrast to these, the cells treated with Colchicine and Doxorubicin supplemented DMEM showed poor morphological features along with signs of cell death.

## 4 Discussion

As stated previously, there are several methods available for performing cell migration studies; however, they suffer from serious drawbacks such as limited reproducibility, destruction of cell monolayer and/or ECM secreted by cells, mechanical or thermal stress, and stress related genetic and/or biochemical changes in cells. Commercial cell inserts that were meant to overcome these drawbacks are very costly and applicable for one time use only. Therefore, we have designed and 3D printed cell inserts with arguably superior characteristics. For 3D printing, we selected high temperature resin to yield the sturdy and autoclave compatible cell inserts. Among the two latest state of the art cell inserts meant for studying cell migration, one provides simple experimental setup owing to its small size and simple design. However, it does not allow for visualization of cells under a microscope in the presence of cell inserts (Acosta et al., 2022). On the other hand, the second bulky cell inserts offer the possibility of cell visualization, but this requires the use of extra devices such as well plate locking and stabilizing clamps (Boyer et al., 2018). Without these clamps, the cell inserts may not fit inside plate and may move during handling. This could lead to compromised reproducibility in the cell migration assays. The use of additional clamps with the inserts makes the procedure cumbersome for high throughput screening. Also, such extra devices increase overall 3D printing cost. By contrast, our cell insert; due to the “plus sign” design at the top that creates a four-quadrant space, allows for microscopy cell visualization in real time, while the insert is kept in the well plate. The “plus sign” design on the top and the “cylindrical” design below it in our cell inserts allow the user to replace the cell culture medium over the course of an experiment. Our inserts need no additional “holding” equipment owing to their simpler design and smaller size. Hence, we would like to argue that our cellinserts are easier to use and more suitable for reproductible and high throughput cell migration assays with real time monitoring. They also allow for exploring additional experimental parameters via easy change of the growth medium. No significant differences in the viability of cells grown in presence of these inserts as compared to control group proves that our inserts are perfectly compatible with A549 cells. Unlike the methods used to create cell-free area such as laser treatment, mechanical stamping, scratching with pipette tip, our cell inserts did not seem to alter the basic physiology of human cells. This guarantees that the findings of studies with our inserts would be solely the result of the cellular response to the medium supplemented with various chemicals. After confirming zero leakage under cell inserts and uniformity of cell-free areas in all wells, we validated them with cell migration study under various treatments. But, before actual cell migration analysis, it is imperative that the image analysis should be hassle-free, easy, and automated for several images. To enable that, we created a macro script in ImageJ platform. Our macro script yielded comparable results (in terms of determination of percent cell-free area) with another available ImageJ program called “Wound healing size tool” (Suarez-Arnedo et al., 2020). Our macro script proved to be superior than “Wound healing size tool” because unlike this tool, it allowed uniform analysis of hundreds of images in one go.

In actual cell migration assay, for validation of these inserts, we used various treatments. Among these treatments, DMEM, FBS, and EGF had positive impacts on cell migration; whereas colchicine and doxorubicin had negative impact. The DMEM supplies basic nutrients (amino acids, mineral salts, and vitamins) for growth and proliferation of cells whereas medium supplemented serum provides additional nutrients such as growth factors, proteins, immunoglobulins, and several small molecules (Kwon et al., 2016). FBS is the most widely accepted serum supplement for cell culture experiments. Owing to availability of growth factors from serum, cells proliferate and migrate with faster rate filling the cell-free area, as it is evident from Figure 5. EGF is a well-known growth factor that promotes and regulates growth and proliferation of primary cells and immortalized cell lines (Kim et al., 2015; Ohnishi et al., 2017). Its positive effect on cell migration; and especially in case of A549 cell line, has been well documented (Lauand et al., 2013; Castro-Aceituno et al., 2016). EGF binds to Epidermal Growth Factor Receptor (EGFR) and show several growth promoting actions such as improved cell proliferation, cell growth and cell differentiation, improved ECM synthesis and ECM remodeling, increased protein synthesis and protein phosphorylation, and increased cell migration (Lauand et al., 2013; Kim et al., 2015; Ohnishi et al., 2017). Accordingly, EGF-supplemented cells migrated faster than those given only DMEM and FBS. Colchicine, originally used as an anti-inflammatory agent, is a microtubule binding alkaloid with potent anticancer activity (Wang et al., 2019). Its exact anticancer mechanism is not yet fully known. It is believed to bind with microtubules and destabilize the microtubule network in the cell (Wang et al., 2019; Oh et al., 2022). Disturbed microtubule network impairs cell migration. Colchicine also induces caspase-3-mediated apoptosis via suppressing the PI3K/Akt/mTOR signaling pathway in some cancer cell lines (Huang et al., 2015; Balkrishna et al., 2019; Zhang et al., 2019; Oh et al., 2022). Specifically in A549 cell line, it binds strongly with microtubule assembly (reported binding constant 0.5 µM), depolarizes the assembly, induce ROS and p53 signaling pathway, and increases proapoptotic to antiapoptotic protein ratio that ultimately leads to cell death (Bhattacharya et al., 2016). Doxorubicin is a known FDA approved anticancer drug that binds with DNA and hinders its repair via topoisomerase-II-enzyme (Thorn et al., 2011; Bojko et al., 2019; Kciuk et al., 2023). It also induces oxidative stress that leads to cell membrane damage, inactivation of proteins, and DNA damage. Even though doxorubicin does not have a direct impact on microtubule assembly, it reduces cell migration probably via inhibition of protein synthesis and prolyl 4-hydroxylase enzyme activity (Thorn et al., 2011; Bojko et al., 2019; Kciuk et al., 2023).

Thus, all these results (Figure 5; Supplementary Figures S1–S3), altogether further strengthen our findings of cell viability assay, where we claimed that presence of our inserts does not affect cellular physiology and cell viability. Rather, cells only respond to given treatment and this observation makes our cell inserts perfectly suitable for high throughput cell migration experiments. Overall, our novel cell inserts yielded highly reproducible results for cell migration study of A549 cell line in presence of known standards such as EGF, colchicine, and doxorubicin. In presence of these agents, the obtained results are similar to what reported in literature for other cancer cell lines, which validate these novel inserts.

## 5 Conclusion

Our overall results suggest that these novel cell inserts provide a simple, fast, and highly reproducible method for cell migration analysis. These inserts are compatible with autoclaving, and with performing high throughput experiments. Due to the special design, these inserts address the main drawbacks of previous devices, namely, they allow real time monitoring of the progressing cell monolayer with a brightfield microscope, easy exchange of medium without interrupting the experiment, and very simple operation, suitable for inexperienced users. Moreover, the newly developed very simple ImageJ macro script allows rapid analysis of thousands of images in reproducible and error-free way. Based on the presented data, we propose that our cell insert design coupled with the new image analysis method provides a clear technological advancement in the field of 2D cell migration studies in terms of simplicity, reproducibility, and robustness.

## Data Availability

The original contributions presented in the study are included in the article/Supplementary Material, further inquiries can be directed to the corresponding author.
