# Supplementary material for: 3D printed inserts for reproducible high throughput screening of cell migration
Source: Front Cell Dev Biol. 2023 Aug 30;11:1256250. doi: 10.3389/fcell.2023.1256250 (PMC10498783; doi:10.3389/fcell.2023.1256250)
Supplement: Supplementary file 1 [file Presentation1.pdf]

frontiers

Supplementary Material

3D printed inserts for reproducible high throughput screening of cell migration

Abhayraj S. Joshi[1], Mukil Madhusudanan[1], Ivan Mijakovic[1,2*]

*** Correspondence:**

Prof. Ivan Mijakovic.
ivan.mijakovic@chalmers.se

1 Method S1: The image analysis using newly developed macro script.

```
run("8-bit");
run("Set Scale...", "distance=282 known=800 unit=µm global");
run("Find Edges");
setAutoThreshold("MaxEntropy dark");
//run("Threshold...");
//setThreshold(82, 255);
setOption("BlackBackground", true);
run("Convert to Mask");
run("Invert LUT");
run("Erode");
run("Fill Holes");
run("Analyze Particles...", "size=100000-700000000 show=[Overlay Masks] display summarize
overlay add");
```

Instructions to use:

1. Open ImageJ/FIJI program.
2. Click on ‘Process’ menu → ‘Batch’ option → ‘Macro’ option.
3. Enter ‘Input’ folder location and ‘Output’ folder location. Input folder is the one where RGB image files are stored; whereas output folder is the one where binary images will be stored.
4. Copy-paste above given macro script in black space.
   (Note: Make sure to change cyan-colored highlighted areas of macro script as per the necessity. For example, the image scale should be fixed as per the magnification of microscope used for imaging.)

5. Click on 'Process' and results will be summarized in a separate window. Save these results in csv format.

2 Method S2: Use of CellTracker software for determining average cell velocities.

We used CellTracker program (Piccinini et al., 2015) to analyze the cell movement direction as well as average cell velocities under all given treatments. For the same, first all the time lapse images were combined to prepare a stack with total of 9 frames (each frame denoting 12 h period) using FIJI software (*Images→Stack→Images to stack*). The stacks were converted to 8-bit grayscale format and then cropped to focus one corner of cell-free area along with some clear cellular structures. Then, a stack of images was opened in CellTracker program. All the procedure for '*Vignetting Correction*' and '*Automatic Alignment*' was performed as instructed by CellTracker developers (Piccinini et al., 2015). We selected '*Manual Tracking*' option. We chose '*Linear Interpolation (Faster)*' mode. We also fixed '*Maximum Cell Displacement*' value to 600 and '*Cell Diameter*' value to 20. After that, we selected 10 different cells at random and tracked their migration in each frame to generate X-Y coordinates. Finally, by clicking on '*Statistics*' menu, we determined cell displacement, average cell velocities, and cell movement directions. As given in figure S1 (A-E), the colored and numbered lines indicate X and Y coordinates of randomly selected cells across 9 frames (Z-axis). It is clear that the cell movement is very high (denoted by erratic wavy line) in EGF treatment (Figure S1-C), intermediate (denoted by lesser erratic wavy lines) in FBS treatment (Figure S1-B) and low (denoted by even lesser erratic wavy lines with some straight lines) in DMEM treatment (Figure S1-A). In case of Colchicine and Doxorubicin treatment, the cell movement was the lowest or none (represented by constant and straight colored lines) (Figure S1-D and E). This validates our cell migration kinetics data. The bar graph (Figure S1-F) shows average cell velocities (µm/h) for given treatments. EGF supplemented and serum supplemented DMEM showed statistically significant and higher cell velocities (One-way ANOVA, p-value < 0.05) as compared to other treatments.

3 Method S3: Use of CellTracker software for determining average cell velocities.

In order to compare the effect of individual treatment with the respective control, we analyzed the cell migration results using two-way ANOVA test. The results obtained from treatment-wise comparison (Figure S2) suggests that during the initial 24 h no significant cell migration changes (in terms of filling up the cell-free area by cell proliferation and cell migration) were observed for any treatment groups. However, at 48 h, cell proliferation and cell migration were faster in EGF treated cells compared to DMEM (without serum), colchicine, and doxorubicin treated cells. At this time point, no significant difference was observed in the migration of cells treated with serum supplemented medium and EGF supplemented medium. After 48 h, a rapid decrease in % cell-free area denotes synergistic action of EGF with FBS, which is evident from figure S2 as two-way ANOVA test showed significant difference in % cell-free area for EGF treatment compared to all other groups past this time point.

4 Method S4: Use of CellTracker software for determining average cell velocities.

Finally, we also checked the cell morphology for given treatments. From bright field images as provided in figure S3, in case of DMEM, DMEM supplemented with 10% FBS, and DMEM supplemented with serum and EGF, the cells looked healthy with normal epithelial morphology. By contrast, the cells treated with colchicine and doxorubicin showed poor morphological features. Very few cells showed epithelial cell like morphology. For majority of cells, the shape was either oval or polygonal. After 24 h, the majority of treated cells showed oval morphology with destroyed cell membranes indicating cell death due to the action of colchicine and doxorubicin (Thorn et al., 2011, Wang et al., 2019, Oh et al., 2022). All these images showed change in morphology owing to the treatment with various supplements and now because of our 3D printed cell inserts with which cell monolayer was grown.

5 Supplementary Figures

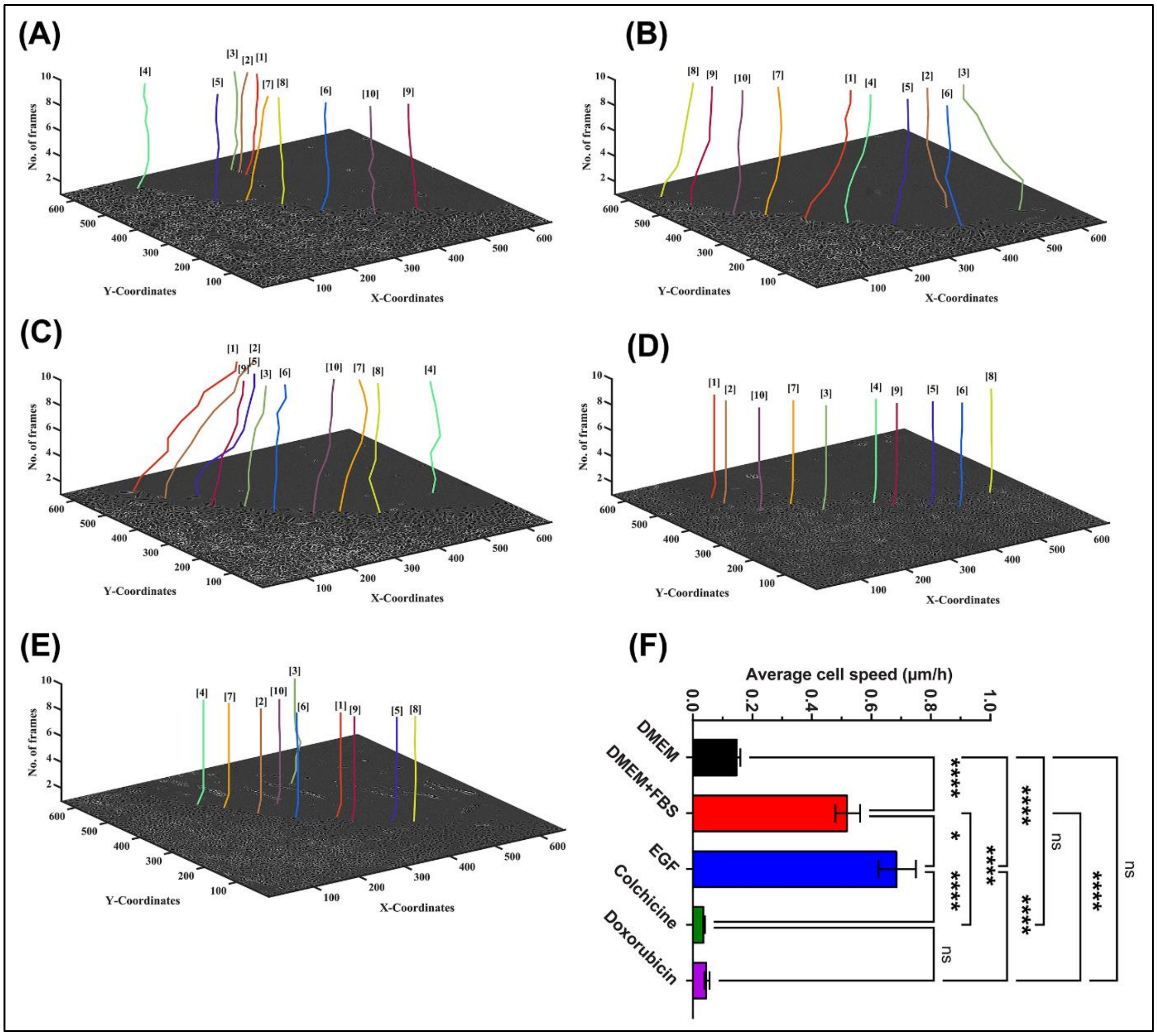

Supplementary Figure 1. Migration pattern and the velocities for A549 cells: **(A-E)** CellTracker data for A549 cells treated with DMEM, FBS, EGF, colchicine, and doxorubicin; where the X-axis and Y-axis denotes the X-Y coordinates for a selected cells during tracking and Z-axis denotes the

number of frames representing time. The brightfield grayscale image at the base of each graph represents position of cell at initial time (t=0); whereas the colored and numbered lines represent migration path of selected cells, **(F)** The bar graph represents average cell speed (*n*=10) in terms of µm/h for given treatment.

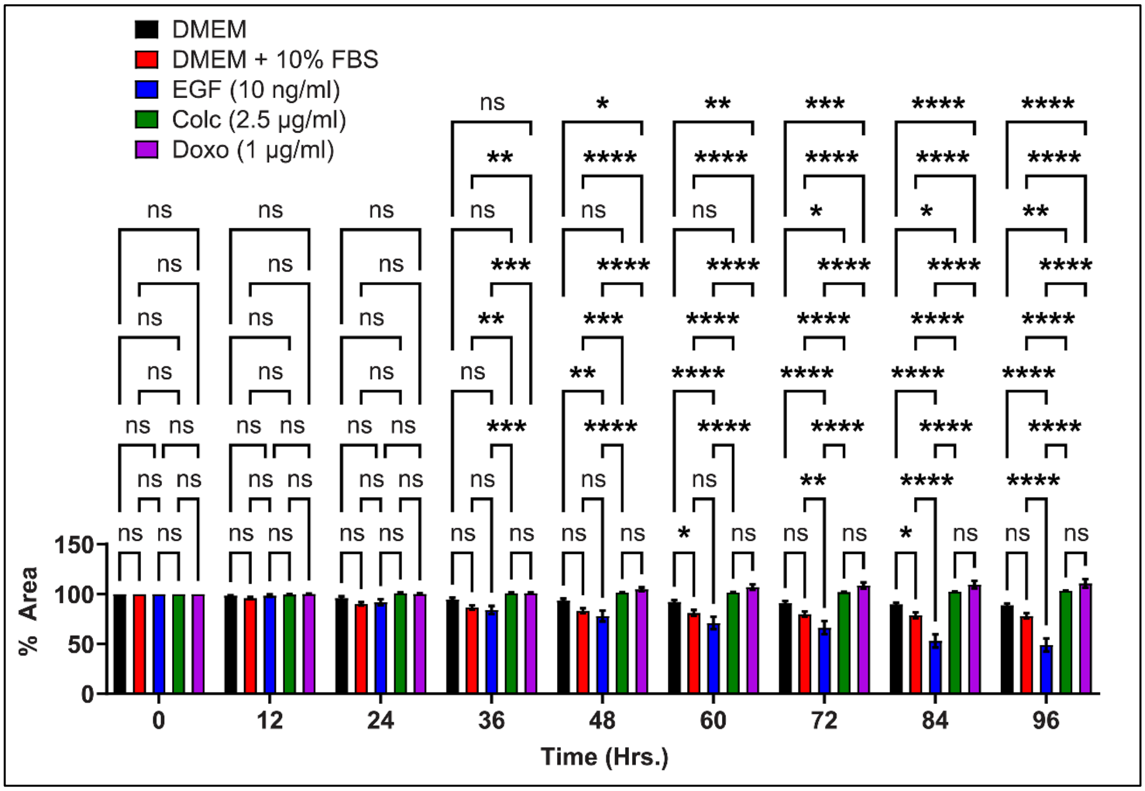

Supplementary Figure 2. Comparative treatment-wise analysis at respective time intervals for A549 cell migration data.

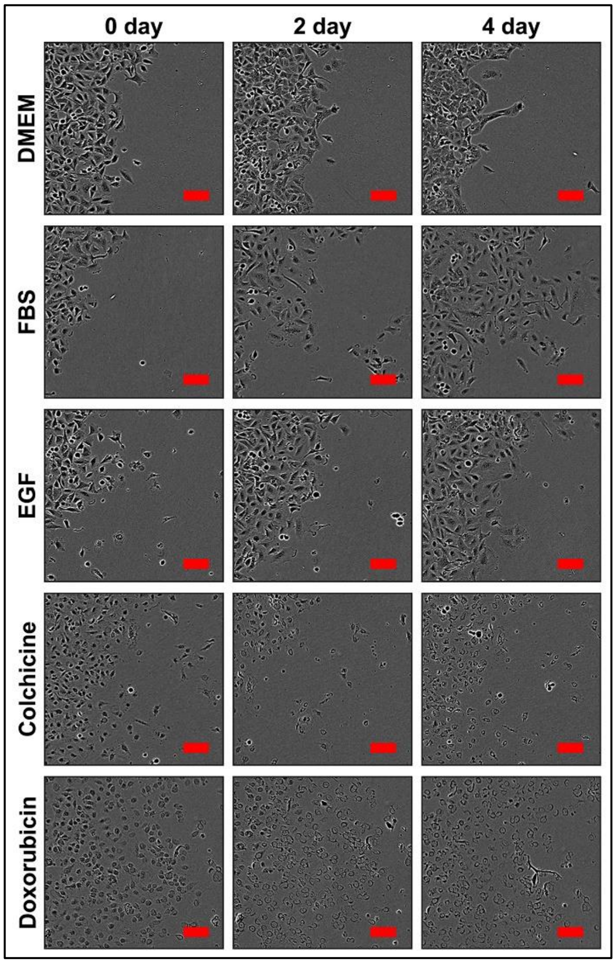

Supplementary Figure 3. Bright field images of A549 cells show differences in cell morphology after treatment with DMEM, FBS, EGF, colchicine, and doxorubicin, (Scale bar = 400 µm).

6 References

OH, J., AN, H. J., YEO, H. J., CHOI, S., OH, J., KIM, S., KIM, J. M., CHOI, J. & LEE, S. 2022. Colchicine as a novel drug for the treatment of osteosarcoma through drug repositioning based on an FDA drug library. *Frontiers in Oncology*, 12.

PICCININI, F., KISS, A. & HORVATH, P. 2015. CellTracker (not only) for dummies. *Bioinformatics*, 32**,** 955-957.

THORN, C. F., OSHIRO, C., MARSH, S., HERNANDEZ-BOUSSARD, T., MCLEOD, H., KLEIN, T. E. & ALTMAN, R. B. 2011. Doxorubicin pathways: pharmacodynamics and adverse effects. *Pharmacogenetics and Genomics*, 21**,** 440-446.

WANG, X., DECKER, C. C., ZECHNER, L., KRSTIN, S. & WINK, M. 2019. In vitro wound healing of tumor cells: inhibition of cell migration by selected cytotoxic alkaloids. *BMC Pharmacology and Toxicology*, 20**,** 4.
